# Dr. Scudamore's Essay on the Blood

**Published:** 1824-06-01

**Authors:** 


*An Essay on the Blood, comprehending the chief Circumstances which influence its Coagulation; the Nature of the Buffy Coat; with a concise medical View of the State of the Blood in Disease; and an Account of the Powers of a saturated Solution of Alum, as a Styptic Remedy in Hæmorrhage.* By Charles Scudamore, M.D. F.R.S. Member of the Royal College of Physicians, &c. &c. Octavo, pp. 162. London, 1824.

Dr. Scudamore, is entitled to much praise for the zeal which he has manifested in these experiments, amounting to nearly one hundred, on the blood—experiments which, as he observes, must have been attended "with peculiar difficulty and trouble." "It may," says Dr. S. "be called a path of labour more useful than agreeable, except as the search after truth always carries with it some reward."

There can be no doubt that an acquaintance with the appearances and actual condition of the blood in disease, must be an important guide in medical practice. How far the *pathology* of the blood may be improved by the experiments which Dr.

Scudamore has made, we shall not at present venture to determine.

"In the treatment of active inflammation, we are directed in the most essensial points, by the symptoms and particular state of the patient; but, even here, we may learn much from the characters of the blood; and in the management of chronic inflammation, and some obscure forms of disease, in which the repetition of venesection becomes doubtful we derive, from a knowledge of the nature of the blood, a very material assistance to our judgment." *Pref.* iv.

The experiments are so very numerous, diversified, and circumstantial, that any attempt to convey an idea of them would be perfectly useless; we must therefore pass at once over one hundred pages, and as many experiments, to our author's "general conclusions and medical observations," from which we shall extract as much as our narrow limits and the nature of the subject will permit.

Although the action of heat in promoting, and of cold in retarding the coagulation of the blood, was pointed out by Hewson, that gentleman does not appear to have been aware of the great power of the former agent in accelerating coagulation. Dr. Scudamore's experiments authorize him, he thinks, to conclude that the period of time in which the blood coagulates, depends, in a great measure, on the quick or slow extrication of the carbonic acid gas. Its evolution takes place most freely as the blood begins to concrete, and ceases when coagulation is complete.

We shall pass over our author's observations on the theory started by Sir Everard Home respecting the formation of blood-vessels by the extrication of carbonic acid from the blood. All we shall say is, that the results of Dr. Scudamore's experiments were "remarkably in opposition" (no uncommon event among experimenters) to those of Sir Everard.

"It may be stated as a general rule, that blood coagulates in the shortest time, accordingly as it is of high specific gravity. Such is the blood of a strong and healthy person, as abounding most in red particles, which constitute the heaviest part of the blood. Mr. Hunter, in speaking of the red globules, remarks that "their use would seem to be connected with strength, for the stronger the animal the more it has of the red globules." The fibrin also belonging to such healthy blood is more dense than that of blood in disease, and hence the real explanation of a quick coagulation.

"The very marked difference of time in which the blood coagulates, accordingly *as the stream from the orifice is fast or slow*, appears to me to warrant the conclusion which I have stated, that the commencing part of the process of coagulation, namely, the escape of carbonic acid, does take place more readily when the blood flows very slowly.

"It appears that *rest*, merely, does not assist the coagulation of the blood. We see in how remarkable a manner cold delays coagulation of the blood in the basin; and how slowly it takes place when confined in a vessel of the living animal. Comparatively, also, as shown in Experiment xxxvi, the blood which was left perfectly at rest, coagulated more slowly than that portion which was gently stirred." 113.

If the blood be received into a metallic vessel the coagulation is slow, from the material being a free conductor of caloric; and the blood, separating more gradually into its component parts in the order of their specific gravity, will more probably exhibit the buffy coat than when a porcelain cup is used.

"In attempting to explain the coagulation of the blood, I should be induced to say that it depends on a new condition of the fibrin, which can only exist fluid in a state of intimate mixture with red particles, serum, and carbonic acid gas. Except in circulation or in living vessels, the fibrin will not maintain an union with the serum, although it does continue blended with the red particles, either when extravasated in the living body, or when taken from the vessels. It is therefore the property of the fibrin to become solid when separated from the several principles just stated, constituting in union the mass which we call blood." 115.

It is found that sizy blood abounds more in serum than healthy blood—but the large quantity that appears (according to Dr. S.) is partly owing to its being forced out from the clot by the strong contraction of the fibrin. In such blood we find the coagulum distinguished by inverted edges, swimming in a large quantity of serum. It thus floats, because the proportion of fibrin, which is of the least specific gravity, exceeds the usual proportion which it bears to the red particles.

The buffy appearance of the blood has been usually accounted for by the slow coagulation of the lymph permitting the red globules, which are much larger and heavier, to sink before that process has taken place. But the fact most difficult of explanation is the variable proportion of fibrin in different cups of blood drawn at the same bleeding—sometimes a difference of more than half between the contents of two cups. It is in inflammation of the fibrous textures of the body that the greatest quantity of fibrin is found in the blood—for example, in inflammation of the heart, pleurisy, and acute rheumatism.

"I am led, therefore, to the idea, that, under such circumstances, the fibrin, instead of being distributed to the fibrous textures as usual in health, remains in excess in the blood. The textures in question would be injured by receiving their usual supply of fibrin, being now in a state of disease; and as regards the variation in the quantity of fibrin in different portions of blood drawn at the same time, it does not appear to me a difficult supposition that in the very short space of time occupied in venesection, the state of circulation should change, so that the capillary arteries at once make a different distribution of the fibrin; resuming in great measure their ordinary economy, and conveying it to the fibrous textures instead of returning it in unnatural excess to the venous circulation. The fact itself of the different proportions of fibrin in different cups of blood filled at the same venesection, is clearly proved. I know not any more probable theory to offer in explanation." 120.

We think the term theory is not quite applicable to the above—it is no more than a hypothesis.

We must refer our readers to Dr. Scudamore's work for his criticisms on Mr. Hunter's favourite doctrines respecting the life of the blood. Its coagulation and other changes out of the body are maintained by Dr. S. to depend on chemical more than vital causes. The influence, however, of the living principle in parts having contact with blood, is very manifest. Thus, if a leech survive its suction, and be examined several weeks afterwards, the blood will be found grumous, but free from signs of decomposition, and will coagulate on being exposed.

Out of its proper vessels the blood coagulates in the living body, as in the cellular membrane, or in an aneurismal sac—and also in the vessels themselves, when they have lost their life.

"We now arrive at the conclusion, that in all circumstances when the blood is in vessels forming part of the living body, even in animals which are torpid during the winter season, it is fluid; but coagulates, or maintains a disposition to coagulate, when the living power of the vessels has ceased, unless the putrefactive process has taken place more or less." 130.

We must pass over some chemical considerations, in order to devote more space to the medical.

In drawing blood by venesection, the physician's attention should be directed to the kind of orifice, state of the pulse, density, colour and other appearances of the blood, its time of coagulation, the texture of the coagulum, and quality of the serum.

When the blood flows slowly, the coagulation takes place so soon, that the fibrin unites itself with the red particles very uniformly, and therefore does not present any appearance of the buffy coat, unless the blood be remarkably sizy.

"I cannot, however, refrain from taking notice of a more important point as regards the stream of blood, namely, its remedial influence on disease. In venesection for the relief of inflammation, our object is not simply to diminish the quantity of circulating blood; but to diminish the action of the heart; and it may be affirmed, that twelve ounces of blood drawn so quickly as to produce an immediate and decided effect on the pulse, will prove much more useful than a considerably larger quantity stolen away so slowly that the heart accommodates itself more easily to the loss. In a case of plethora, or apoplectic congestion, the same reasoning does not so exactly apply, because the absolute quantity of blood to be drawn is more to be considered. Invariably, however, a good orifice is important; and hence, it is of great consequence that the operation of venesection should be well performed." 137.

To a certain extent and no farther, we agree with our author and many practitioners on the above points. We have watched pretty narrowly the phenomena and effects of venesection; and the result of our observations is, the conviction that some practitioners attach too much importance to the effect of *syncope* in checking inflammation. We are convinced, that if syncope take place before a considerable quantity of blood be withdrawn, in an internal inflammation, the object will not be gained, and reaction will come on as violent as ever. We therefore rarely or never order a patient to stand up while being bled, with the view of more quickly inducing faintness, but let that take place while the patient is sitting or recumbent. We repeat it, that unless a considerable quantity of the vital fluid be withdrawn, the induction of syncope will have but a very temporary effect on the inflammation of an internal organ, and that, by such a procedure, no economy of blood is effected in the end. We are advocates, of course, for a free issue of the blood through a proper orifice, though we look upon the rule laid down by the late Dr. Pemberton, and quoted by Dr. Scudamore (eight ounces in three minutes) as a mere refinement, worthy only of people who affect astonishing nicety and sagacity in the most trifling things. No two people bleed alike, however large or small may be the orifices, and therefore rules like the above are quite inapplicable to clinical practice.

We agree with Dr. Scudamore that, in dangerous inflammations, and especially peritoneal inflammation, the finger should be kept on the pulse, and the flow of blood allowed to continue, whatever the quantity, until hardness and frequency be distinctly reduced.

From the colour of the blood while issuing from the veins, little, we think, of a practical nature, is to be gained. In quick respiration, as in phthisis pulmonalis, the blood is florid; but if there be much difficulty of transmission through the lungs, it is generally of a very dark hue.

"The blood should be received in different cups; and the breakfast cup is the most suitable size. They should be put by in order, all in the same situation as to temperature, and not disturbed, so that we may be enabled to draw some useful inference of the effect produced on the circulation during the operation of venesection, by the relative appearances of the different portions of blood. In deciding upon the mere presence or absence of the fibrinous coat, we must particularly refer to the quick or slow stream in which the blood has flowed. In active inflammation of the fibrous texture, the formation of the buffy coat cannot be prevented; because the fibrin in the blood is in such excess; but when inflammatory action of the circulation does not much prevail, we shall learn more of the nature of blood, from examining its texture, than from merely viewing the surface of the coagulum. I consider that the texture of the coagulum and its degree of contraction indicate more of the actual state of the heart and arteries, than the mere presence or absence of the fibrinous coat. In the examination of blood taken away by cupping, we have to form our judgment chiefly from the texture of the coagulum, as to the eye it almost constantly presents an uniform appearance. In general terms, I may state, that a firm texture of the blood points out a strong action of the blood vessels, so as to give a presumptive sign that the bleeding has been proper; and vice versâ, if the coagulum be remarkably loose in texture, we should particularly question the propriety of repeating the operation. I may further remark, that when the clot possesses uniform firmness, and has its edges turned inwards, we may conclude that the blood vessels act more strongly than when it is soft in its texture throughout, and having a thin and flabby edge. The extreme toughness of the buffy coat itself, and this contrasted with the soft texture of the inferior part of the coagulum, is matter of familiar observation." 143.

In respect to the buffy coat, as indicative of inflammation, Dr. S. remarks, that an increased velocity of the circulation *alone* will not produce this phenomenon, as proved by experiment. It requires the continuance of disease to give rise to the buffy coat. It will not be found in blood drawn in the first few hours of inflammatory action. It is to be expected in diseases accompanied by much wasting, as in diabetes. In pregnancy it may depend on the same cause, for the rest of the body generally wastes while the uterine system is increasing. At the same time that buffy blood generally indicates inflammatory action in the system, no one surely would think of drawing blood, or continue to bleed from this symptom *alone*. The judicious practitioner will take all circumstances into considertion when he orders the detraction of blood.

Perhaps the most important part of this essay yet remains to be noticed, though it occupies but a few pages; namely Dr. Scudamore's Observations on a Saturated Solution of Alum as a Styptic to Bleeding Vessels. In conjunction with Mr. Wood, Dr. S. made experiments with this and other substances on the wounded arteries of a dog, and the result gave the palm of victory to the alum.

"The sulphate of copper, and still more Ruspini's styptic, had an effect upon the hæmorrhage proceeding from very small arterial branches, but not the slightest upon an artery of considerable size, which bled vehemently. On pouring the alum solution on this vessel, we were equally surprised and gratified to witness, that in about a quarter of a minute, the bleeding entirely ceased; and that it was thus effectually restrained, by means of the tenacious coagulum so promptly formed, operating as a plug to the mouth of the vessel, was proved several times, by sponging away the coagulum, seeing the violent renewal of the hæmorrhage; repeating the application with perfect success; and so on." 157.

Our author has had some opportunities of witnessing the efficacy of this styptic applied to the human subject. In one case, where a carbuncle was laid open, the hæmorrhage could not be stopped till a saturated solution of alum was applied. The same was the case in a wound of the temporal artery by a scarification. The artery was divided but still the hæmorrhage continued, in spite of various styptics. The saturated solution quickly arrested the flow of blood. Some other cases are related.

The solution,* Dr. S. remarks, should be applied warm, as being still more coagulating than when cold, agreeably to the experiments detailed in his work. In the mean time, cold may easily be applied to the neighbouring parts, in order to diminish vascular action.

"In some cases of hæmorrhage from the unimpregnated uterus, and especially of the passive kind, I apprehend that the free injection of this solution would be attended with the most important advantage; and indeed I am sanguine enough to think, that, under certain circumstances of hæmorrhage after delivery, and the removal of the placenta, it will promptly prove a remedy for a state of imminent danger, when other means would probably fail. I should recommend the injection to be used tepid, by means of a proper and efficient apparatus; while, at the same time, if the urgency of the case should require such an additional step, cold might be employed externally on the abdomen, in the usual manner.

"In conversation with my friend Mr. Clarke, of Saville Row, I received the high sanction of his opinion, that the treatment, suitably applied, would be very proper. He does not apprehend that in the case of uterine hæmorrhage, any risk of mischief would arise from the free injection of this solution." 160.

Alum, it is well known, has been employed for centuries, as

* One ounce of cold distilled water holds completely in solution 31 grains of alum—and infusion of roses 34 grains.

a styptic—but not in the form of a saturated solution, on which our author thinks its whole merit depends. Dr. S. has given the remedy, with success, internally, in a case of hæmoptysis, and in another of hæmatemesis. The infusion of roses with mucilage of acacia and a little syrup was the vehicle. The alum was taken in free doses, without any inconvenience to the stomach. Dr. S. will feel obliged to his professional brethren for any communication respecting the effects of this styptic (favourable or unfavourable) which may be founded in experience.

